# TRPC3 and TRPC6: Multimodal Cation-Conducting Channels Regulating Cardiovascular Contractility and Remodeling

**DOI:** 10.3390/cells15020144

**Published:** 2026-01-14

**Authors:** Takuro Numaga-Tomita, Motohiro Nishida

**Affiliations:** 1 Department of Molecular Pharmacology, Shinshu University School of Medicine and Health Sciences, Matsumoto 390-8621, Japan; 2 Division of Cardiocirculatory Signaling, National Institute for Physiological Sciences (NIPS), National Institutes of Natural Sciences, Okazaki 444-8787, Japan; 3 Exploratory Research Center on Life and Living Systems (ExCELLS), National Institutes of Natural Sciences, Okazaki 444-8787, Japan; 4 Department of Physiology, Graduate School of Pharmaceutical Sciences, Kyushu University, Fukuoka 812-8582, Japan

**Keywords:** TRPC3, TRPC6, cardiovascular system

## Abstract

Transient receptor potential canonical (TRPC) channels function as multimodal cation channels that integrate chemical and mechanical cues to regulate cellular signaling. Among them, TRPC3 and TRPC6 have been studied primarily in the context of cardiovascular and renal physiology, and their roles in other organ systems are now increasingly recognized. Although these channels are known to be activated downstream of phospholipase C (PLC) signaling, especially 1,2-diacylglycerol (DAG) production, their precise modes of activation under native physiological conditions remain incompletely understood. Recent structural and functional studies have greatly advanced our understanding of their primary activation by DAG. This review summarizes how decades of physiological analyses have revealed multiple modes of TRPC3 and TRPC6 channel activation beyond DAG gating, providing a broader perspective on their diverse regulatory mechanisms. This review also highlights recent progress in elucidating the channel properties, activation mechanisms, and the physiological as well as pathophysiological roles of TRPC3 and TRPC6 in cardiovascular contractility and remodeling, and discusses the remaining challenges that will lead to the establishment of TRPC3 and TRPC6 as validated therapeutic targets.

## 1. Introduction

Over the past decades, transient receptor potential (TRP) channels have emerged as versatile cellular sensors that detect a wide variety of physical and chemical stimuli. Since the advent of high-resolution TRP channel structures by cryo-electron microscopy in recent years, structural biology has provided unprecedented insights into their gating and regulatory mechanisms, complementing decades of physiological and biochemical studies. This review focuses on TRPC3 and TRPC6, whose activation mechanisms and physiological roles have been illuminated largely through studies in the cardiovascular system, with additional mechanistic insights from renal research. We also summarize accumulating evidence that positions TRPC3/6 channels as promising therapeutic targets in cardiovascular disease.

### 1.1. Transient Receptor Potential (TRP) Channels

The *trp* gene was originally identified in *Drosophila* based on a mutant phenotype that impaired visual transduction [1]. This spontaneous mutation only produced a transient receptor potential when exposed to continuous light. In mammals, 28 homologues of TRP channels have been described, which are categorized into six major subfamilies according to sequence and functional similarities: TRPC (canonical), TRPV (vanilloid), TRPM (melastatin), TRPP (polycystin), TRPML (mucolipin), and TRPA (ankyrin) [2]. Despite their diversity, TRP channels share common structural motifs, including six transmembrane segments, a pore loop between the fifth and sixth segments, and a conserved 25–amino acid stretch known as the “TRP domain” [3]. The TRPC subgroup, consisting of seven mammalian isoforms (TRPC1–TRPC7), is considered the closest evolutionary counterpart to the original Drosophila channel [4]. Consistent with this, they were proposed and subsequently confirmed to operate as calcium ion (Ca^2+^)-permeable non-selective cation channels downstream of surface receptor activation. Within this family, TRPC4 and TRPC5 exhibit approximately 65% sequence identity [5,6], whereas TRPC3, TRPC6, and TRPC7 share about 75% homology [7,8]. The difference in homology correlates with the functional difference among TRPC channels, that is TRPC3/6/7 are activated by lipid second messenger diacylglycerol (DAG) but TRPC4 and 5 are not [9].

### 1.2. General Characteristics of TRPC3/6/7 Channel

TRPC3/6/7 channels share the general structural features of TRP channels, including N-terminal ankyrin repeats, six transmembrane segments with a pore-forming loop between the fifth (S5) and sixth (S6) segments, and a C-terminal EWKFAR motif known as the “TRP box.” All TRPC channels are activated downstream of phospholipase C (PLC)–coupled receptor stimulation. PLC hydrolyzes phosphatidylinositol 4,5-bisphosphate (PIP_2_) into inositol 1,4,5-trisphosphate (IP_3_) and 1,2-DAG. Activation of PLC elicits Ca^2+^ entry into the cell via two major pathways: store-operated and receptor-operated. Store-operated Ca^2+^ entry (SOCE) is induced by endoplasmic reticulum (ER) Ca^2+^ depletion through IP_3_-mediated IP_3_R activation [10]. The molecular components of SOCE are now known to be the ER Ca^2+^ sensor STIM1 and the plasma membrane (PM) channel Orai1 [11,12,13,14,15,16]. Before their discovery around 2006, TRPC channels were considered the best candidates for store-operated channels. Although pure SOCE is mediated by STIM1 and Orai1, TRPC channels can be activated secondarily by rises in intracellular Ca^2+^ and thus contribute to Ca^2+^ influx in a broader sense [17]. However, the precise roles of TRPC channels in SOCE remain elusive. TRPC3/6/7 channels are non-selective cation channels characterized by an outwardly rectifying current–voltage relationship with a reversal potential near 0 mV. Their relative Ca^2+^/Na^+^ permeability ratios (P_Ca/P_Na) are approximately 1.6, 5, and 2 for TRPC3, TRPC6, and TRPC7, respectively [18]. The pore region contains a highly conserved LFW motif, mutations of which completely abolish channel function [19,20].

### 1.3. Structural Insights into DAG-Mediated Activation Mechanisms of TRPC3 and TRPC6

As mentioned above, activation of PLC produces not only the soluble second messenger IP_3_ but also the membrane-restricted messenger DAG. In 1999, Hofmann et al. demonstrated that the membrane-permeable DAG analog 1-oleoyl-2-acetyl-sn-glycerol directly activates TRPC3 and TRPC6 [9]. Although this DAG-induced activation has been consistently reproduced, the underlying gating mechanism of these channels remained elusive for decades. Recent advances in structural biology, however, have started to reveal the molecular basis of DAG-mediated channel activation. Activation of ion channels is generally mediated by direct lipid–protein interactions or indirect mechanisms such as changes in channel-surrounding membrane properties [21,22]. Lipid-dependent modulation is commonly observed in TRP channels, best exemplified by the modulatory effect of phosphoinositides [23]. Earlier computational analysis predicted a DAG binding site in the N-terminal TRP_2 domain of TRPC3 [21]. However, the association of DAG at this domain is required for the membrane fusion of TRPC3-containing vesicles. Therefore, this interaction could not account for the DAG-mediated gating of TRPC3. Furthermore, splice variant analysis of TRPC6 revealed that the N terminus (residues 3–56) is crucial for activation of TRPC6 by DAG [24], although subsequent studies could not reproduce this result [25].

Recent homology modeling, molecular dynamics (MD), and mutational analyses of TRPC3 identified key gating elements and suggested that lipid binding may allosterically couple to the permeation pathway [26]. Importantly, these models predicted potential lipid recognition sites at fenestrations within central transmembrane segments of the tetramer, highlighting structural gaps as possible entry points for lipophilic ligands such as DAG [27]. By combining homology modeling with an optical ‘lipid clamp’ strategy using the photo-switchable DAG analog OptoDArG, recent work identified a fenestrated subunit interface in TRPC3 as a critical site for DAG sensing [28]. A glycine residue (G652) located behind the selectivity filter was shown to govern both gating flexibility and lipid recognition. The G652A mutation modified the channel’s DAG discrimination and altered the kinetics and cooperativity of activation, supporting the concept that DAG activates TRPC3 by binding within pore-domain fenestrations that are allosterically coupled to gating [28]. The glycine residue is well conserved among TRPC channels. The conserved glycine in the pore-domain fenestration (G652 in TRPC3, G709 in TRPC6) is essential for DAG-dependent gating. Thus, both channels possibly share a conserved DAG-sensing machinery, although the precise impact on lipid recognition may differ between subtypes [28]. Although the glycine residue is highly conserved across TRPC channels, the surrounding residues required for interaction with DAG differ between TRPC3/6/7 and TRPC4/5 [29].

**Figure 1:**
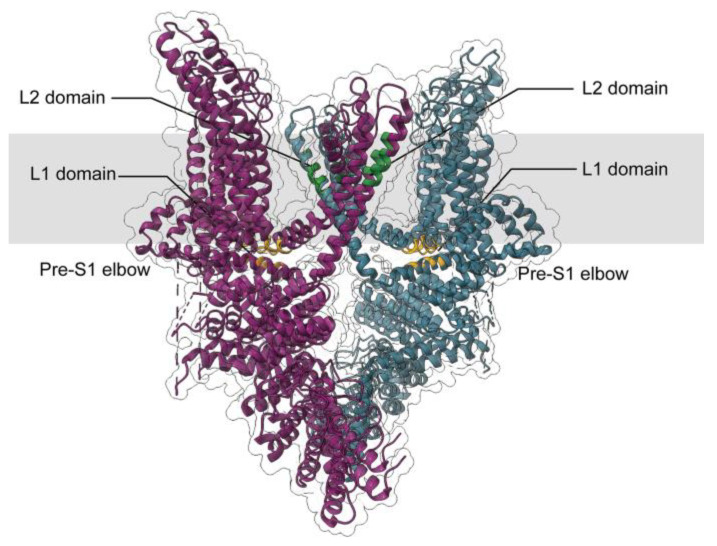
Positions of the lipid-like density (L) domains within the TRPC3 channel. Structural model based on the cryo-EM structure (PDB: 6CUD). Two diagonal subunits of the tetramer are shown in magenta and cyan. Residues forming the L1 and L2 domains are colored in yellow and green, respectively. The putative transmembrane region is shaded in gray. The structure is oriented so that the Pre-S1 elbow regions extend laterally, providing a side view parallel to the membrane plane.

Building upon these predictions, the subsequent cryo-electron microscopy (cryo-EM) analysis of full-length human TRPC3 provided the first structural framework for understanding lipid recognition and channel gating. This structural advance, together with later MD and photo-pharmacological studies, offered direct experimental validation of the proposed lipid-sensing mechanism. The cryo-EM structure of full-length human TRPC3 (hTRPC3) was first reported in 2018 [30]. This structure (PDB: 6CUD) captured the channel in a lipid-occupied, closed state at 3.3-Å resolution. The ion-conducting pore adopted a firmly closed conformation, with the narrowest radius being less than 1 Å near I658 and L654 on the S6 helix, effectively preventing ion passage. Despite being closed, the structure revealed two key lipid-like densities within the transmembrane domain (TMD), termed lipid-like density 1 (L1) and lipid-like density 2 (L2). The L1 site is formed by a pocket between the pre-S1 elbow and the S4–S5 linker and is thought to be closely linked to channel activation due to its interaction with S4 and the S4–S5 linker (Figure 1). The L2 site is in the lateral fenestration of the pore domain, wedged between the P loop and S6 of the adjacent subunit (Figure 1). It is in close contact with the conserved LFW motif and is considered a critical determinant of lipid recognition in TRPC3 [19,20]. Importantly, this fenestrated L2 pocket corresponds closely to the lipid entry site previously predicted by homology modeling and mutational analyses, providing a structural validation of earlier functional hypotheses [27,28]. Since the resolved cryo-EM structure of TRPC3 is the closed state of the channel, the coexistence of lipid densities at both the L1 and L2 sites may suggest that these pockets are occupied by inert phospholipids rather than DAG in the closed state, as also proposed by subsequent studies.

Subsequent study using MD simulations and photo-pharmacological approaches demonstrated that the closed state of TRPC3 serves as a functional platform for DAG capture and channel sensitization, which can be further modulated by other phospholipids and cholesterol [31]. It also indicated that DAG rapidly accumulates within the TMD of TRPC3, preferentially at the L2 site, due to its intrinsic hydrophobicity and ability to flip between membrane leaflets. Even at low concentrations, DAG partially occupies multiple L2 sites within the closed tetramer, establishing a pre-lipidated state that facilitates subsequent channel activation.

The importance of DAG interaction with closed TRPC3 channels was revealed by repetitive optical stimulation using the photo-switchable DAG analog OptoDArG [31]. This approach uncovered a characteristic sensitization phenomenon: the second and subsequent activations of the channel occurred significantly faster than the initial one. The acceleration of activation kinetics indicates that DAG interaction with TRPC3 can induce a sensitized closed state even while the channel remains non-conductive. In this state, DAG lipidation of the L2 coordination site occurs without opening the permeation pathway, thereby priming the channel for subsequent activation [31]. Mutagenesis studies confirmed the pivotal role of the L2 pocket, particularly through the G652A mutation, whereas mutations in the L1 site produced no comparable effects. In addition, the G652A mutation underscores the structural precision of lipid sensing in TRPC3 through the L2 site.

Collectively, these findings provided the basis for a two-step activation model of TRPC3 by DAG [31]. In this model, partial sub-stoichiometric DAG lipidation of the L2 sites establishes a primed, sensitized state that facilitates faster activation upon renewed DAG exposure. At higher DAG concentrations, further lipidation of the remaining L2 sites likely promotes additional conformational rearrangements, culminating in full pore opening. Structural analyses further suggest that while the initial activation requires concerted rearrangements of all four subunits, only two subunits need to undergo subsequent structural changes for the final opening of sensitized channels [31]. Nonetheless, full activation of TRPC3 may also involve additional DAG interactions outside the L2 site, which cannot be excluded at this stage. In this context, it is noteworthy that mutagenesis studies have revealed a cluster of basic residues within the pre-S1 elbow, located in close proximity to the L1 site of TRPC6, as a binding site for PIP_2_ [32]. Depletion of PIP_2_ is known to negatively regulate TRPC3/6/7 activity [33,34]. Importantly, PIP_2_ binding to this region is independent of DAG binding, providing strong evidence that lipid interactions near the L1 site—distinct from the L2 site—play a critical role in controlling TRPC6 channel activity. Although clusters of basic residues are generally conserved among TRPC channels, the specific basic residue motif (K/RXXK/RXXRXXXXK) is uniquely conserved in the TRPC3/6/7 subfamily [32]. Consistent with this prediction, a PIP_2_ binding site has been identified in TRPC3 at the pre-S1–proximal L3 site, which is structurally homologous to the pre-S1 elbow/shoulder in TRPC6, indicating a conserved lipid-sensing module within this subfamily. Notably, in TRPC3, PIP_2_ binding at the L3 site is essential not only for receptor- or DAG-induced activation but also for sustaining the constitutive channel activity that characterizes this channel [35].

These observations have established a structural framework for understanding how DAG binding at the L2 pocket and PIP_2_ interactions within the pre-S1–proximal region adjacent to the L1 site drive and regulate TRPC3/6 activation through conformational coupling. However, accumulating evidence indicates that DAG-dependent gating represents only one facet of TRPC3 and TRPC6 function, which can also be modulated by store-dependent and context-specific mechanisms discussed below.

### 1.4. Multimodal Activation of TRPC3 and TRPC6: Revisiting Store-Dependent Regulation

Historically, TRPC3 was once regarded as a potential component of SOCE, a view supported by its activation following ER Ca^2+^ depletion. However, the identification of STIM1 and Orai1 as the core molecular machinery of SOCE led to the gradual exclusion of TRPC channels from this paradigm. Classical studies demonstrated that heterologous expression of TRPC3 in either HEK293 or COS-1 cells increased thapsigargin (TG)-induced SOCE, suggesting that TRPC3 might constitute part of the SOC channel complex [36,37,38]. However, later studies reinterpreted these results and showed that the increased TG-induced Ca^2+^ entry largely reflected the constitutive activity of TRPC3 channels [39]. Before the discovery of STIM1 and Orai1, the store depletion-induced activation of TRPC3 or TRPC6 was thought to occur via their coupling with IP_3_ receptors [36]. Indeed, both TRPC3 and TRPC6 possess binding sites for either IP_3_ receptors or calmodulin (CaM) in their C-termini, termed the CaM- and IP_3_R-binding (CIRB) domain [40,41,42]. The N-terminal TRPC3-binding domain of IP_3_R increased the basal activity of TRPC3, indicating that coupling with IP_3_R positively regulates TRPC3 channels [43]. Yet, one of the most critical findings from these early studies has often been overlooked—the dependence of SOCE-like activation on TRPC3 expression levels [44]. The discovery of STIM1 and Orai1 newly shed light on the possible involvement of TRPC3 in the SOC channel complex. Although a direct interaction with STIM1 has been demonstrated for TRPC1/4/5 but not for TRPC3/6/7 [45,46], suggesting that the latter channels can be activated independently of STIM1, their interaction with Orai1 [47] and hetero-multimerization with TRPC1 [48,49] may indirectly couple TRPC3/6 to STIM1-induced SOCE. Thus, TRPC3/6/7 could be activated downstream of IP_3_-mediated Ca^2+^ release. As mentioned above, the activation of TRPC3 depends on its expression level, and recent studies on endogenously expressed TRPC3 and TRPC6 have introduced a new layer of complexity to our understanding of their activation mechanisms.

The advent of RNAi and genetic knockout technologies has made it possible to dissect the contribution of endogenous TRPC3 and TRPC6 under physiological conditions. A growing number of studies have demonstrated that these channels physically and functionally interact with STIM1, IP_3_Rs, and other components of the ER–plasma membrane (PM) junctions, thereby participating in store-dependent Ca^2+^ signaling [50]. In endothelial cell lines, TRPC3 silencing diminished TG-induced Ca^2+^ increases, possibly via the PLC/PKCε pathway [51]. Interestingly, linoleic acid-induced DAG production activates TRPC3 in taste bud cells, and this effect is completely abolished by STIM1 knockdown [52], suggesting that STIM1 plays a critical role in TRPC3 activation even by DAG. This interaction can be explained by the observation of Liu et al. [53]. According to their study, STIM1 serves as the principal tether required for the formation of ER–PM junctions associated with PM PIP_2_. Within these PIP_2_-enriched domains, receptor activation brings G protein–coupled receptors (GPCRs) and TRPC channels into close proximity, enabling effective receptor–channel communication and multiple modes of PIP_2_-dependent regulation [32,33,34]. PIP_2_ not only serves as the precursor for DAG that activates the channel but also binds to L1 site to inhibit TRPC3 opening and regulate access of DAG to pore L2 site, while influencing channel selectivity and localization at ER–PM junctions [53]. Consistent with this concept, TRPC3 has been shown to functionally couple with IP_3_ receptors within ER–PM nanojunctions, where local Ca^2+^ entry through TRPC3 shapes IP_3_R-dependent Ca^2+^ oscillations and enables switching between distinct spatiotemporal Ca^2+^ signaling modes [54]. STIM1 is also reportedly important for PM expression of TRPC6 and its hetero-multimerization with TRPC1/3 [55]. The component of SOC channel varies in cells. It has been shown that STIM2 specifically gates the neuronal SOC (nSOC) pathway in postsynaptic spines. [56]. STIM2 is a homolog of STIM1 that serves as a more sensitive sensor of ER Ca^2+^ levels, becoming partially activated under resting conditions to maintain basal Ca^2+^ influx [57]. In hippocampal neurons, TRPC6 forms a complex with STIM2 and Orai2 which functions as an nSOC channel. In this complex, TRPC6 serves as a Ca^2+^-conducting channel and ER Ca^2+^ levels are transduced from Orai2 by means of association with STIM2 [58]. The coupling of STIM2 and TRPC6 has been found in the breast cancer MCF7 cells [59]. These data raise the possibility that the distinction between store-operated and receptor-operated activation of TRPC3/6 is not clear-cut. Jardin et al. proposed that the interaction with Orai1 determines the participation of endogenous TRPC6 in SOC entry, whereas its association with TRPC3 under Ca^2+^-dependent conditions mediates receptor-operated Ca^2+^ entry [60]. This dynamic switching of interaction partners—between the Orai1–STIM1 complex and TRPC3—suggests that TRPC6 acts as a molecular hub linking receptor stimulation to distinct Ca^2+^ entry pathways. Consistent with this view, we have demonstrated that native TRPC3 functions as a receptor-operated Ca^2+^-permeable channel in the PM, serving as a signaling hub that recruits PLCγ and PKCβ to amplify Ca^2+^ and DAG signaling in DT40 chicken B lymphocytes [61]. This DAG-dependent signal amplification persisted even in the absence of all three IP_3_Rs, indicating that TRPC3 functions independently of SOCE. These findings underscore the context-dependent gating versatility of TRPC3 and TRPC6. Their mode of activation—store-operated or receptor-operated—appears to depend on the cellular and signaling milieu, as well as on the stoichiometry of homo- and hetero-tetrameric assemblies that have yet to be fully defined.

Although the contribution of TRPC3/6-containing SOC channels to cardiovascular physiology appears to be limited, several studies have nevertheless reported their involvement in distinct cardiovascular contexts. In cardiomyocytes, TRPC3 contributes to pathological hypertrophic remodeling downstream of cardiovascular risk factors and neurohumoral stimulation [62]. Nicotine induces sustained TRPC3-mediated Ca^2+^ influx, leading to activation of the calcineurin–NFAT signaling pathway and subsequent upregulation of TRPC3 expression, thereby establishing a positive feedback loop that amplifies prohypertrophic Ca^2+^ signaling [63]. In vascular endothelial cells, TRPC3 functions as a key regulator of Ca^2+^-dependent nitric oxide (NO) production. TRPC3-mediated Ca^2+^ entry is required for ATP-induced activation of endothelial NO synthase, contributing to both transient and sustained Ca^2+^ signals that underlie endothelial responsiveness to chemical and mechanical stimuli [64]. In vascular smooth muscle cells (VSMCs), TRPC3 is preferentially expressed and supports SOCE that promotes cell cycle progression and proliferation. Genetic or pharmacological suppression of TRPC3 markedly inhibits VSMC proliferation, induces cell cycle arrest, and attenuates neointima formation in vivo, while relatively preserving endothelial repair [65]. Beyond the vascular wall, TRPC3 expression in immune cells is enhanced under high-salt conditions and correlates with increased SOCE, daily salt intake, and systolic blood pressure in patients with essential hypertension, suggesting that TRPC3 may act as a salt-sensitive regulator linking immune cell Ca^2+^ signaling to blood pressure control [66]. Collectively, these findings highlight a contribution of store-dependent TRPC3 activation to cardiovascular remodeling and homeostasis.

As several studies have reported altered expression patterns of individual TRPC channels under pathological conditions [67,68,69,70,71], understanding how the composition of endogenous TRPC3/6 complexes changes between physiological and disease states remains a major unresolved question—one that may hold the key to their diverse functional repertoires.

## 2. Mechanosensitive Activation and Physiological Relevance of TRPC3/C6

In the preceding sections, we have focused on the mechanisms by which TRPC3 and TRPC6 are activated downstream of PLC signaling. Beyond their well-established roles in receptor-mediated pathways, both channels are recognized as key players in the cardiovascular system—a highly dynamic environment in which mechanical forces serve as critical regulators of cellular function. In addition to their PLC-dependent activation, TRPC3 and TRPC6 can also be stimulated by mechanical cues. This section highlights the molecular basis and physiological relevance of their mechanically induced activation.

The activation of TRPC6 channel by mechanical stimuli was first demonstrated in cerebral arterial smooth muscle cells which was treated with hypo-osmotic solution. The exact mechanism of mechanical activation of TRPC6 remains elusive. However, several models are proposed: (1) TRPC6 itself senses the stretch of lipid surrounding the channel which is independent of PLC activity when overexpressed in CHO cells and in murine cardiomyocytes. Important to note, activation of TRPC6 in these studies was sensitive to mechanosensing channel inhibitor GsTMx4 [22,72]. However, later studies denied the direct activation of TRPC6 by membrane stretch [73,74,75]. (2) TRPC6 couples to G_q_-coupled GPCR such as Angiotensin II (Ang II) type I receptor (AT_1_R) which senses the membrane stretch and transforms the signal into PLC activation [73,76]. (3) TRPC6 is not directly activated by mechanical stimuli. Instead, TRPC6 becomes mechanosensitive only after receptor stimulation, with 20-hydroxyeicosatetraenoic acid (20-HETE), an arachidonic acid–derived vasoconstrictive lipid mediator, likely acting as a critical determinant [77]. Subsequent work demonstrated that TRPC6 channels localized within cholesterol-rich lipid rafts are sensitized, as this microenvironment imposes a higher baseline membrane tension. Under such conditions, receptor activation produces a more pronounced channel response compared with cholesterol-depleted membranes, indicating that membrane tension within lipid rafts positively modulates receptor-dependent TRPC6 activation [78]. This elevated membrane tension within lipid rafts can be explained by their role as mechanical hubs at the PM. Lipid raft domains preferentially anchor the actomyosin cytoskeleton, thereby concentrating intracellular contractile forces and transmitting them to raft-localized membrane proteins, including TRPC6 [78]. Importantly, although PIP_2_ is distributed across both raft and non-raft regions, phosphoinositide turnover and DAG production proceed with markedly faster kinetics within lipid rafts [79]. In addition, the ordered lipid environment of raft domains restricts the trans bilayer flip-flop of DAG, thereby stabilizing its preferential accumulation in the cytoplasmic leaflet [80]. Together, these mechanical and kinetic properties position lipid rafts as platforms that integrate membrane tension with localized DAG signaling to fine-tune receptor-dependent TRPC6 activation. Recently MD simulation also predicted cholesterol binding site within TRPC3. These simulations indicate that cholesterol binds dynamically to both annular and non-annular sites in TRPC3, including a non-annular site at the interface between the voltage-sensor like domain and pore domains, where it may stabilize the transmembrane structure and the domain-swapped tetrameric topology independently of membrane phospholipid composition [81], further suggesting the structural importance of membrane rafts for TRPC3/6 channel function. These findings suggest that receptor activation and mechanical stress are not independent but interdependent mechanisms that synergistically modulate TRPC6 function through reciprocal regulation of membrane tension and signaling dynamics. However, most studies relied on TRPC6 overexpression in heterologous expression systems such as HEK293 or CHO cells. While overexpression of TRPC channels is useful for dissecting minimal channel activation mechanisms and facilitates statistical analysis, native mechanical environmental changes are transduced through complex molecular machinery that involves not only the lipid membrane but also cytoskeletal components, such as the actin cytoskeleton, and focal adhesion complexes.

**Figure 2:**
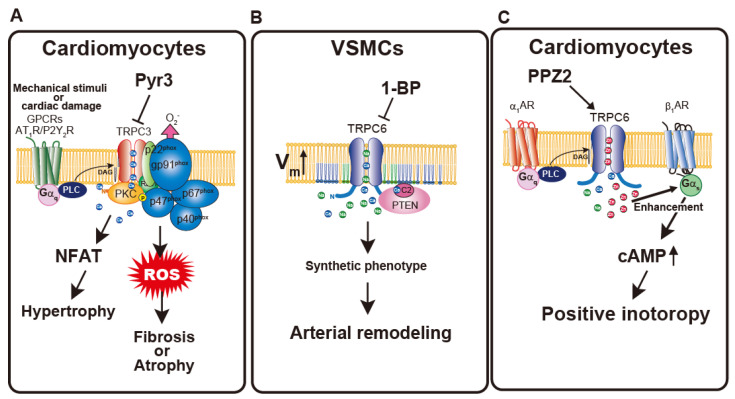
Multimodal regulation of TRPC3 and TRPC6 channels in cardiovascular pathophysiology and their therapeutic modulation. (**A**) In cardiomyocytes, mechanical stress or cardiac damage activates G_q_-coupled GPCRs such as AT_1_R and P2Y_2_R, leading to PLC activation, DAG production. This signaling activates TRPC3, which forms a scaffold for the PKC and NOX2 complex and promotes NFAT activation, ROS production, and subsequent hypertrophic as well as fibrotic or atrophic remodeling. The TRPC3 inhibitor Pyr3 suppresses TRPC3–NOX2 coupling and thereby attenuates ROS-dependent cardiac damage. (**B**) In VSMCs, TRPC6-mediated cation influx depolarizes the membrane potential (Vm↑) and modulates C2-domain–containing PTEN, maintaining VSMCs in a synthetic phenotype that drives arterial remodeling. The TRPC6 inhibitor 1-BP blocks this pathway and facilitates a shift toward a more differentiated, contractile phenotype, thereby limiting pathological vascular remodeling. (**C**) In cardiomyocytes, TRPC6 is activated downstream of α_1_-adrenergic receptor (α_1_AR)–G_q_–PLC–DAG signaling and can also be directly stimulated by the TRPC6 activator PPZ2. TRPC6-mediated Zn^2+^ influx enhances β_1_-adrenergic receptor (β_1_AR)–Gs–cAMP signaling, resulting in increased cAMP production and positive inotropy, thus supporting cardiac contractile function under stress conditions.

Subsequent studies employing gene silencing and primary cells isolated from TRPC3- and TRPC6-knockout mice have shed light on the mechanisms underlying mechanical activation of TRPC3 and TRPC6, as well as their physiological relevance. Seo et al. demonstrated that TRPC6 channels in cardiac muscle cells are directly activated by mechanical stretch, contributing to stress-stimulated contractility (SSC); this mechanosensitive response is significantly attenuated in TRPC6 knockout TRPC6^−/−^ mice or by TRPC6 inhibitors, highlighting TRPC6 as a key mediator of cardiac mechano-transduction [82]. Like TRPC6, TRPC3 also contributes to the mechanical stress-induced increase in Ca^2+^ levels in cardiomyocytes [83,84]. Sustained stretching of cardiac muscle leads to a gradual elevation of intracellular Ca^2+^ levels over several minutes, known as the stress-induced slow Ca^2+^ increase (SSC). TRPC3 knockout in cardiomyocytes abolished this response. In this context, however, TRPC3 does not directly sense membrane stretch; instead, mechanical activation of the AT_1_R triggers PLC signaling, which subsequently activates TRPC3. Beyond this physiological setting, TRPC3 also plays an important role under pathological stretch conditions. Pathological stretch of cardiomyocytes, which frequently occurs in failing hearts, activates TRPC3, which in turn activates NADPH oxidase 2 (NOX2), whose activity exacerbates cardiac dysfunction (Figure 2A). This stretch-induced, NOX2-dependent ROS production is abolished in TRPC3-knockout hearts [85]. TRPC3 not only provides Ca^2+^ required for NOX2 activation but also acts as a stabilizing partner that protects NOX2 from degradation. Mechanical activation of TRPC6 plays a crucial role in maintaining myogenic tone in cerebral arteries, as TRPC6 knockout completely abolishes pressure-induced constriction in these vessels [86]. Hypo-osmotic stress induces a non-selective cation current, and TRPC6 silencing abolishes this current in human pulmonary arterial endothelial cells [87]. These studies clearly demonstrate the involvement of TRPC3 and TRPC6 in mechanosensitive signaling within the cardiovascular system. However, as discussed above, it remains unclear whether mechanically induced activation of TRPC3/6 operates independently of neurohumoral factor-induced activation, because in the native cardiovascular environment these inputs are likely to be highly interdependent, as suggested by earlier observations of mechanically mediated sensitization of receptor-dependent signaling [77,78].

The distinction between receptor-mediated and mechanically induced activation has been clarified by observations in non-cardiovascular systems. Ca^2+^ imaging of intact kidney glomeruli demonstrated that intraglomerular Ca^2+^ increases evoked by glomerular capillary pressure were strongly suppressed in TRPC6^−^/^−^ mice, suggesting that TRPC6 functions as part of the mechanosensing machinery in the glomerulus [88]. Anderson et al. demonstrated that membrane stretch induced by hypo-osmotic stress or direct mechanical stimulation through perfusion or physical touch activates endogenously expressed TRPC6 channels in mouse podocyte cell lines [89]. Importantly, podocin, a membrane protein essential for maintaining podocyte structure, acts as a molecular filter that distinguishes between mechanical (stretch-induced) and chemical (DAG-induced) activation of TRPC6 channels, providing direct evidence that these two modes of activation are mechanistically separable [89]. The balance between these two modes of activation strongly depends on both the abundance and functional integrity of podocin. The same group later identified a TRPC6 missense mutation (Asn143Ser) causing familial focal and segmental glomerulosclerosis, which selectively abolished mechanical activation without affecting DAG-induced activation, thereby again revealing a clear mechanistic distinction between the two activation modes [90]. Although the involvement of TRPC3 and TRPC6 in cardiovascular physiology and pathophysiology is well established, non-cardiovascular systems have provided important insight into how the intensity of mechanical stimuli influences TRPC-dependent signaling. In periodontal ligament stem cells, TRPC6 activation induced by appropriate mechanical force contributes to periodontal tissue reconstruction by regulating cell migration and bone remodeling. Importantly, the contribution of TRPC6 is most evident under moderate mechanical loading, whereas excessive mechanical stress diminishes its regulatory impact, suggesting that additional, TRPC6-independent mechanisms dominate under conditions of severe mechanical strain [91]. In a related example, studies in renal epithelial cells demonstrated that distinct mechanical stimuli engage distinct TRP channels, with hypotonic cell swelling preferentially activating TRPC3. Importantly, TRPC3-dependent Ca^2+^ signaling is coupled to adaptive regulation of cell volume, suggesting that TRPC3 functions not only as a transducer of mechanical cues but also as a component of mechano-regulatory feedback [92]. This observation is conceptually consistent with stretch-mediated activation of TRPC3 reported in pathological cardiac remodeling, in which disruption of mechano-regulatory negative feedback mechanisms is thought to facilitate prolonged TRPC3 activation and maladaptive cardiac remodeling. Taken together, accumulating evidence indicates that TRPC3 and TRPC6 participate in mechanosensitive Ca^2+^ signaling not as passive force sensors, but as components of regulatory circuits tuned to specific mechanical contexts. Insights from non-cardiovascular systems further suggest that TRPC3 and TRPC6 can be activated through mechanistically distinct pathways, including receptor-mediated and mechanically induced modes, and that these channels function within defined mechanical ranges and adaptive feedback loops, the disruption of which may underlie pathological cardiac remodeling.

## 3. Beyond the Ca^2+^ Channel: Physiological Implications of TRPC3/C6 as Non-Selective Cation Channels

As described so far, TRPC3 and TRPC6 channels have attracted attention primarily as channels mediating Ca^2+^ influx. However, this represents only a part of their functional repertoire. Muscle cells express voltage-dependent L-type Ca^2+^ channels (LTCCs), which exhibit higher selectivity for Ca^2+^ and greater conductance. Therefore, TRPC3/6 channels are thought to mediate cation influx in response to vasoconstrictor stimulation, which induces membrane depolarization and subsequently activates LTCCs. Under such conditions, it remains uncertain whether Ca^2+^ entry through TRPC3/6 channels produces distinct cellular outcomes, or whether such effects can be clearly distinguished from those mediated by LTCCs or other Ca^2+^-mobilizing pathways. In non-excitable cells, by contrast, the absence of dominant voltage-gated Ca^2+^ entry makes it easier to discriminate among different Ca^2+^ sources, such as DAG-activated influx, IP_3_R-mediated release, and SOCE, each of which may contribute to distinct signaling pathways.

Membrane potential is a fundamental biophysical property that differs among cell types and contributes to diverse cellular functions [93]. Its importance has recently attracted renewed attention, as subtle variations in resting potential are increasingly recognized to influence signaling sensitivity and cell behavior. The fact that TRP channels are not strictly Ca^2+^-selective but rather function as non-selective cation channels strongly suggests that they also play key roles in the regulation of membrane potential. While membrane potential is primarily determined by the activity of K^+^ channels, which set the resting potential and repolarization dynamics, recent studies have highlighted that cation influx through non-selective channels, acting as a counterbalance to K^+^ efflux, also plays a crucial role in maintaining or fine-tuning the membrane potential and thereby modulating cellular responsiveness [93]. We and others have reported such regulation of membrane potential by TRPC6 [94,95]. VSMCs change their phenotype between quiescent, differentiated contractile and highly proliferative, migratory synthetic states to maintain vascular integrity. As mentioned above, TRPC6 contributes to LTCC activation by depolarizing the membrane potential in response to vasoconstrictive stimuli. However, the expression of proteins involved in the contractile machinery is reduced in synthetic VSMCs. Interestingly, in VSMCs, the resting membrane potential (RMP) of the synthetic phenotype is more depolarized than that of the contractile phenotype [94,96]. We found that TRPC6 activity decreases when cells differentiate into the contractile phenotype, leading to hyperpolarization of the RMP. Notably, the depolarized membrane potential in synthetic VSMCs does not primarily serve to drive downstream Ca^2+^ signaling. Instead, it activates Phosphatase and Tensin Homolog Deleted from Chromosome 10 (PTEN), a negative regulator of the Akt pathway that promotes VSMC differentiation toward the contractile phenotype (Figure 2C) [94]. The phenotypic switching of VSMCs from a contractile to a synthetic state involves re-entry into the cell cycle from a quiescent G0 phase. Evidence for the potential role of TRPC6 in this process has been obtained from studies in bone marrow stromal cells (BMSCs). In these cells, TRPC6 plays a critical role in regulating the RMP by mediating membrane depolarization. This TRPC6-dependent control of RMP directly affects the magnitude of SOCE, which is required for appropriate cell cycle progression and cellular proliferation. Thus, modulation of membrane potential by TRPC6 is pivotal for maintaining the Ca^2+^ influx necessary for cell cycle control [95]. These findings suggest that TRPC6-mediated membrane depolarization regulates not only L-type Ca^2+^ channels but also other Ca^2+^-permeable pathways, thereby fine-tuning Ca^2+^-dependent intracellular signaling processes such as proliferation and cellular differentiation, both of which are central to VSMC homeostasis. These findings redefine membrane depolarization as a bona fide signaling modality, capable of directly regulating intracellular signaling proteins, as previously established for K-Ras signaling [97]. Another intriguing example illustrating the physiological importance of TRPC6 as a non-selective cation channel has been reported. TRPC6 channels translocate to the phagosomal membrane in response to G-protein signaling, where they facilitate cation efflux and help neutralize the membrane potential generated by proton pumps, thereby promoting phagosomal acidification and microbicidal function even in cells lacking functional cystic fibrosis transmembrane conductance regulator [98]. This finding expands the functional landscape of TRPC6 beyond the PM, positioning it as an intracellular cation channel that modulates organellar membrane potential and ion homeostasis. Although intracellular functions of TRPC6 remain underexplored in cardiovascular cells, this work raises the possibility that TRPC6-mediated control of intra-organellar cation flux contributes to ion-dependent signaling pathways with potential relevance to cardiovascular physiology and pathophysiology.

TRPC channels are reportedly permeable to Na^+^, Ca^2+^, K^+^, Cs^+^, Ba^2+^, and Mn^2+^ [99]. These cations are often used experimentally for fluorescence imaging or current recording in patch-clamp studies. Among the TRPC subfamily, TRPC6 has been reported to exhibit unique permeability properties, allowing the passage of trace metal ions such as Zn^2+^ and Fe^2+^ [99].

Zn^2+^ is an essential trace metal ion that serves as a structural or catalytic cofactor for numerous enzymes and transcription factors, thereby playing vital roles in cellular signaling and homeostasis. TRPC6 was identified by Boulon’s group as a Zn^2+^-conducting non-selective cation channel, regulating Zn^2+^ entry in both heterologous expression systems and native cortical neurons [100]. Sustained TRPC6 overexpression leads to intracellular Zn^2+^ accumulation and altered Zn^2+^ homeostasis, resulting in metallothionein induction, increased oxidative stress sensitivity, and changes in cell growth and cytoskeletal organization [100,101]. We have also demonstrated that TRPC6-mediated influx serves as a crucial physiological regulator in the cardiovascular system [102,103]. TRPC6-mediated Zn^2+^ influx was found to potentiate β-adrenoceptor–stimulated positive inotropy in rodent cardiomyocytes (Figure 2C). Deletion of TRPC6 impaired sympathetic nerve-induced positive inotropy, indicating that TRPC6 contributes to baroreflex control of cardiac contractility [102]. Mechanistically, Zn^2+^ influx through TRPC6 enhances α_1_-adrenoceptor–dependent βAR/G_s_ signaling by preventing β-arrestin–mediated βAR internalization (Figure 2C). Pharmacological enhancement of TRPC6-mediated Zn^2+^ influx ameliorated chronic heart failure in mice, suggesting that TRPC6 serves as a physiological amplifier of cardiac sympathetic regulation. As mentioned previously, membrane depolarization resulting from non-selective cation entry through TRPC6 contributes to the maintenance of the synthetic phenotype by activating PTEN (Figure 2B) [94]. Along with depolarizing stimuli, TRPC6 also increases the intracellular Zn^2+^ pool, which supports the proliferative phenotype and contributes to pathological arterial remodeling [103]. Zn^2+^ deficiency in TRPC6^−/−^ mice has also been reported in the placenta, liver, kidney and brain but not in the lung [104].

**Figure 3:**
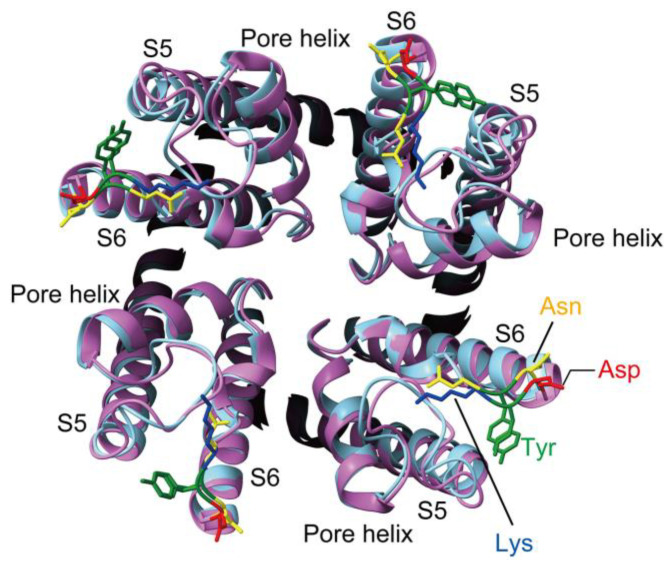
Top-view comparison of the pore region in TRPC3 and TRPC6 Cryo-EM structures of TRPC3 (PDB ID: 5ZBG) and TRPC6 (PDB ID: 5YX9) in the closed state were aligned and visualized using UCSF ChimeraX. A top view along the pore axis is shown, focusing on the S5–pore helix–S6 region that forms the outer pore architecture. TRPC3 is shown in cyan and TRPC6 in magenta. THEKYD motif in TRPC3 and the corresponding NYN motif in TRPC6, located at the extracellular end of S6 near the pore entrance, are highlighted as stick representations. Individual side chains are color-coded (Lys (K), blue; Tyr (Y), green; Asp (D), red; Asn (N), yellow) to emphasize differences in side-chain orientation around the pore mouth. This view illustrates how subtle local rearrangements at the S6–pore interface may reshape the electrostatic and steric environment of the outer pore, potentially contributing to distinct ion permeation and regulatory properties of TRPC3 and TRPC6.

Unlike TRPC6, TRPC3 lacks permeability to Zn^2+^. Comparative analysis of these two channels in the aforementioned study identified key amino acid residues responsible for Zn^2+^ selectivity. These residues are N615YN617 in TRPC6, instead of KYD in TRPC3 and TRPC7, both of which are impermeable to Zn^2+^. Substitution of this NYN motif in TRPC6 with KYD (a TRPC3-like mutation) abolishes its permeability to Zn^2+^. Zn^2+^ is a divalent ion that interacts strongly with charged residues. In TRPC3, the charged KYD motif creates a dense electrostatic environment that hinders Zn^2+^ permeation, whereas the neutral, polar NYN motif in TRPC6 provides a smoother pore surface favorable for Zn^2+^ passage. Cryo-EM comparison between TRPC3 and TRPC6 reveals that this region, connecting the pore loop and the inner S6 helix, plays a crucial structural role in shaping the outer pore entrance and influencing the selectivity filter linkage (Figure 3) [105,106]. Small conformational differences here—especially at the S6–TRP linker—can tilt or widen the pore vestibule, modulating how ions approach and interact with coordinating residues at the entrance of the selectivity filter. TRPC6’s NYN motif helps maintain a less electrostatically restrictive and more open pore geometry, favoring Zn^2+^ permeation, whereas the KYD sequence promotes a tighter, charge-dense pore conformation that disfavors Zn^2+^ transit.

Beyond the cardiovascular system, accumulating evidence indicates that TRPC6 also participates in the regulation of trace metal influx in non-cardiovascular tissues, highlighting a broader role for this channel in metal-dependent cellular processes and pointing toward an as-yet underappreciated relevance in the cardiovascular context. Renal HK-2 cells exhibited TRPC6-mediated Zn^2+^ influx, which protected them against ischemia–reperfusion injury by inhibiting necrosis and promoting autophagy [107]. This protective role of TRPC6-mediated Zn^2+^ influx in ischemia–reperfusion injury suggests possible parallels in ischemic diseases of the cardiovascular system, such as myocardial infarction. Notably, activation of TRPC3/6 has also been reported to protect cardiomyocytes from ischemia-induced cell death [108]. Although the underlying mechanisms remain incompletely defined, these observations raise the possibility that TRPC-mediated Zn^2+^ influx may similarly contribute to cardiomyocyte survival under ischemic stress.

Beyond Zn^2+^ signaling, additional evidence suggests that TRPC6 may be involved in Fe^2+^ influx, a process of particular importance in pathological settings characterized by iron dysregulation and oxidative stress. PC12 cells are a rat pheochromocytoma cell line that differentiates into a neuronal phenotype in response to nerve growth factor (NGF). Mwanjewe and Grover found that NGF-treated PC12 cells take up non–transferrin-bound iron (NTBI) through the TRPC6 channel, whose expression is upregulated by NGF treatment [109]. Activation of TRPC6 by GPCR stimulation or DAG enhances NTBI uptake in heterologously expressed HEK293 cells [109]. Therefore, TRPC6 may constitute a unique mechanism coupling receptor-mediated signaling to the uptake of NTBI, thereby expanding the physiological repertoire of TRPC6 beyond canonical cation entry. This study raises the possibility that TRPC6 may link receptor-mediated signaling to iron handling in cardiovascular cells, particularly under pathological conditions associated with elevated non–transferrin-bound iron and oxidative stress.

## 4. Therapeutic Potential of TRPC3/C6 Modulators for Cardiovascular Diseases

Accumulating evidence from mouse models has progressively highlighted the therapeutic potential of TRPC3 and TRPC6 modulators in cardiovascular diseases. Our laboratory first demonstrated that pharmacological inhibition of TRPC3 with Pyr3 attenuates cardiac dysfunction in both transverse aortic constriction (TAC)-operated mice and MLP^−/−^ mice, a genetic model of hypertrophic cardiomyopathy [110,111]. Pyr3 treatment preserved cardiac contractility while suppressing cardiac hypertrophy and fibrosis in MLP^−/−^ mice. In this context, we uncovered a mechanistic link between TRPC3 and ROS generation, leading to the discovery of TRPC3–NOX2 coupling as a key driver of pathological remodeling. TRPC3–NOX2 coupling contributes not only to cardiac hypertrophy but also to cardiomyocyte atrophy. Doxorubicin (DOX), an effective chemotherapeutic agent, induces dose-limiting cardiotoxicity characterized in part by myocyte atrophy. Pyr3 treatment protected DOX-treated hearts from contractile dysfunction and cell-volume reduction, suggesting that TRPC3 inhibition may help mitigate this major adverse effect [112]. We further demonstrated that atrophy induced by DOX or nutrient deprivation is mediated by ATP released from damaged cardiomyocytes, which activates the P2Y_2_ receptor and subsequently stimulates NOX2-coupled TRPC3 to enhance ROS production [113]. Pyr3 has also been reported to ameliorate myocardial ischemia/reperfusion injury [114]. Pyr3 was originally identified as a pyrazole-based inhibitor with a marked preference for TRPC3 over other closely related TRPC isoforms. In heterologous expression systems, Pyr3 at concentrations effective for TRPC3 inhibition failed to suppress TRPC6 or several TRPM channels [110]. However, subsequent studies revealed that Pyr3 can inhibit STIM/Orai-mediated Ca^2+^ entry [115], indicating that its selectivity is not absolute. Thus, while Pyr3 has been instrumental in demonstrating the therapeutic relevance of TRPC3 inhibition in experimental models of cardiac remodeling, particularly when interpreted alongside complementary genetic and mechanistic evidence, careful interpretation is required in complex in vivo settings where multiple Ca^2+^ entry pathways coexist. Importantly, genetic ablation of TRPC3 provides independent support for these conclusions in both pressure-overload-induced cardiac remodeling and DOX-induced cardiomyocyte atrophy, as TRPC3-deficient mice exhibit resistance to cardiac dysfunction and remodeling, underscoring TRPC3 as a critical pathogenic driver in heart failure [87,116]. Subsequently, Seo et al. demonstrated that selective inhibitors, GSK2332255B and GSK2833503A suppressed pressure-overload-induced cardiac fibrosis. Although both antagonists exert clear suppressive effects on Ang II- or endothelin 1-induced cellular hypertrophy in vitro, their rapid metabolism and high plasma protein binding prevent them from maintaining effective concentrations in vivo, resulting in a failure to suppress cardiac hypertrophy [117]. These findings collectively highlight TRPC3 as a central mediator of ROS-driven pathological remodeling across diverse forms of cardiac stress.

The involvement of TRPC6 in cardiac dysfunction has also been reported [62,118]. Pharmacological inhibition of TRPC6 by larixyl acetate, a selective antagonist derived from larch balsam [119], was shown to reduce TAC-induced contractile dysfunction and cardiac hypertrophy [120]. Genetic and experimental evidence converge to support a pathogenic role of enhanced TRPC6 activity in DOX-induced cardiotoxicity. Genetic deletion of TRPC6 in mice markedly attenuates doxorubicin-induced cardiac damage and preserves cardiac function, particularly in males [121]. In addition, a TRPC6 gene polymorphism, N338S, has been identified as a risk factor for doxorubicin-induced cardiomyopathy; this gain-of-function variant stabilizes DAG binding at the L2 site, thereby enhancing channel activation [122]. Together, these findings indicate that excessive DAG-dependent activation of TRPC6 is a key driver of anthracycline cardiotoxicity and highlight TRPC6 as a potential therapeutic target for cardioprotection in cancer patients. In contrast to these findings, our recent work has demonstrated a cardioprotective role of TRPC6 [70,102]. We have identified two distinct functions of TRPC6. First, TRPC6 exerts a counteracting effect against TRPC3-mediated exacerbation of heart failure by suppressing TRPC3–NOX2 coupling, thereby reducing ROS production [70]. Second, TRPC6 functions as a Zn^2+^-permeable channel whose activation enhances the inotropic effect of β-adrenergic receptor stimulation, helping to maintain cardiac contractility and attenuate adverse remodeling [102]. Consistent with these findings, pharmacological activation of TRPC6 by 2-[4-(2,3-dimethylphenyl)-piperazin-1-yl]-N-(2-ethoxyphenyl)acetamide (PPZ2) [123] markedly restored cardiac contractility in TAC-operated mice, even when administered after contractile dysfunction had already developed [102]. These findings suggest that, beyond traditional inhibitory approaches, activating TRPC6 may also hold therapeutic potential as a new way to support cardiac function under pathological stress.

**Figure 4:**
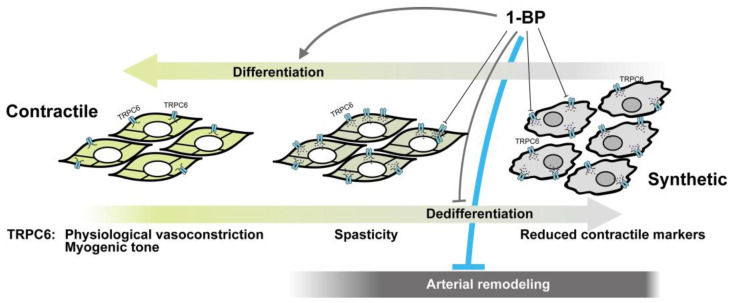
Contribution of TRPC6 to pathological arterial remodeling and its therapeutic potential. Schematic illustration of a continuous process of VSMC phenotype switching and associated TRPC6-dependent functional responses. In the healthy arterial wall, differentiated contractile VSMCs exhibit physiological vasoconstriction and myogenic tone with relatively low TRPC6 activity. Under metabolic and inflammatory stress, arterial remodeling gives rise to a partially dedifferentiated VSMC phenotype that still retains vasocontractile capacity but exhibits increased TRPC6 expression and function, leading to abnormal Ca^2+^ handling and vascular spasticity. With sustained and more severe remodeling stimuli, such as vessel injury accompanied by ischemia, VSMCs undergo complete dedifferentiation into a synthetic phenotype characterized by reduced expression of contractile markers and increased migratory and proliferative capacity, thereby driving arterial remodeling. The color gradient indicates progressive changes in the VSMC phenotype switching process. Pharmacological inhibition of TRPC6 by 1-BP is proposed to suppress maladaptive TRPC6 signaling, plausibly limiting vascular spasticity, while attenuating arterial remodeling and favoring redifferentiation toward a contractile phenotype.

As described in Section 2, TRPC6 plays essential roles in VSMCs. Several studies, including ours, indicate that TRPC6 is a promising therapeutic target in vascular diseases. Idiopathic pulmonary arterial hypertension (PAH) is a progressive and life-threatening disorder characterized by pulmonary arterial remodeling and elevated pulmonary arterial pressure, ultimately leading to right heart failure. TRPC6 expression is reportedly upregulated in pulmonary arterial smooth muscle cells from patients with idiopathic PAH [69,124]. Jain et al. showed that the orally active TRPC6 inhibitor BI-749327 reverses established pulmonary hypertension, with the broad TRP channel blocker 2-APB producing similar improvements, supporting a pathogenic contribution of TRPC6 [125]. BI-749327 was developed as an orally bioavailable TRPC6 antagonist with nanomolar potency and substantially improved selectivity over the closely related TRPC3 and TRPC7 channels. While its activity against several other TRP channels and non-TRP ion channels was reported to be minimal [126]. Beyond PAH, aberrant remodeling of systemic arterial VSMCs is a fundamental pathogenic process in many arterial diseases. As noted in Section 3, TRPC6-mediated depolarization maintains the synthetic phenotype of VSMCs [94]. While VSMC phenotypic switching is essential for vascular repair, excessive proliferation and migration drive disease progression. Thus, promoting the differentiation of synthetic VSMCs toward the contractile phenotype represents an attractive therapeutic concept (Figure 4). Supporting this view, pharmacological inhibition of TRPC6 by the 1-benzylpiperazine derivative (1-BP) facilitates the transition of synthetic VSMCs to the contractile phenotype, and studies using a mouse hindlimb ischemia model demonstrated that 1-BP treatment significantly improves post-ischemic blood flow recovery [127]. Further insight into the stage-dependent involvement of TRPC6 in pathological remodeling is provided by studies in metabolic syndrome (MetS). Li et al. demonstrated that metabolic stress increases TRPC6 together with TRPC1 expression in coronary arteries undergoing atherogenesis, and that enhanced histamine-induced coronary contraction in pigs with MetS depends on increased TRPC6 activity [128]. Importantly, these findings indicate that TRPC6 upregulation occurs prior to complete dedifferentiation of VSMCs, revealing a differential contribution of TRPC6 during the phenotypic switching process in pathological remodeling. In this intermediate remodeling state, partially dedifferentiated VSMCs in the medial layer still retain vasocontractile capacity. Enhanced TRPC6 activity in these cells augments Ca^2+^ mobilization compared with healthy coronary arteries from lean pigs, thereby predisposing the vessel to vascular spasticity (Figure 4). Together, these findings underscore the pivotal role of TRPC6 in pathological vascular remodeling and highlight TRPC6 inhibition as a promising therapeutic approach for arterial diseases (Figure 4).

Despite compelling evidence from multiple mouse models supporting TRPC3 and TRPC6 as therapeutic targets in cardiovascular diseases, several challenges must be addressed before these findings can be translated into clinical applications. One major obstacle is the difficulty in dissociating pathological from physiological channel functions. TRPC3 and TRPC6 are broadly expressed and participate in adaptive signaling under basal conditions, such as vascular tone regulation, raising concerns that sustained or non-selective modulation may compromise essential homeostatic responses. Another challenge arises from the complex and context-dependent roles of these channels. In the heart, TRPC3 primarily acts as a pathogenic driver through Nox2-dependent ROS generation, whereas TRPC6 can exert both deleterious and protective effects depending on disease context, cell type, and mode of activation. This functional divergence suggests that simple channel blockade may not be sufficient, and that disease- and mode-selective modulation—such as targeting TRPC3–Nox2 coupling or selectively enhancing beneficial TRPC6 signaling—may be required. From a pharmacological perspective, the high sequence and structural homology among TRPC family members poses a major challenge for clinical translation [129,130]. Accordingly, the high degree of sequence and structural similarity among TRPC isoforms inherently complicates the development of inhibitors with strict isoform selectivity, and selectivity should therefore be interpreted with caution. Such residual cross-reactivity complicates the interpretation of in vivo effects and raises the possibility that therapeutic outcomes may reflect combined or opposing modulation of multiple channels rather than inhibition of a single pathogenic target. Together, these considerations underscore the need to refine targeting strategies beyond conventional channel inhibition, toward context- and mode-selective modulation, and to better define the pathological settings in which inhibition or activation of TRPC3 or TRPC6 is most likely to confer clinical benefit.

## 5. Conclusions

Over the past two decades, our understanding of TRPC3 and TRPC6 has evolved from initial observations in heterologous expression systems to increasingly sophisticated insights into their structural organization and regulatory mechanisms. While recent cryo-EM studies have provided important frameworks for channel gating and modulation, these structures primarily represent simplified homomeric assemblies and do not fully capture the complexity of TRPC3 and TRPC6 function in native cardiovascular cells. In the cardiovascular system, accumulating evidence indicates that TRPC3 and TRPC6 play context-dependent roles in both physiological adaptation and pathological remodeling. Beyond their canonical function as Ca^2+^-permeable non-selective cation channels, these channels influence cardiac and vascular contractility, membrane excitability, redox signaling, and trace metal homeostasis, thereby shaping stress-dependent remodeling responses in both the myocardium and the vasculature, including hypertrophy, fibrosis, cardiomyocyte atrophy, and vascular smooth muscle phenotypic switching. Importantly, the balance between adaptive and maladaptive TRPC3/6 signaling appears to depend on cellular context, disease stage, and mode of channel activation.

Despite substantial progress, critical gaps remain in our understanding of how native TRPC3 and TRPC6 channel complexes operate in intact cardiovascular tissues and how their multimodal regulation is integrated in vivo. At the same time, recent advances in structural biology, together with MD simulations, are beginning to provide a framework for linking channel architecture to distinct modes of activation and regulation. These emerging approaches offer new opportunities for the in silico design of TRPC modulators that selectively target specific activation modes or channel conformations, rather than indiscriminate pore blockade. Such mode-selective modulation is likely to be particularly informative in vivo, as it may allow dissection of context-dependent TRPC3 and TRPC6 functions under physiological and pathological conditions. Ultimately, integrating structural and computational insights with in vivo models may pave the way toward the development of disease state-specific TRPC3/6 modulators, addressing a major challenge for therapeutic translation in cardiovascular disease.

## Data Availability

No new data were created or analyzed in this study. Data sharing is not applicable to this article.

## Abbreviations

The following abbreviations are used in this manuscript:

Ang II	Angiotensin II
AR	Adrenergic receptor
AT1R	Ang II type 1 receptor
CaM	Calmodulin
cryo-EM	Cryo-electron microscopy
DAG	1,2-diacylglycerol
DOX	Doxorubicin
ER	Endoplasmic reticulum
GPCR	G protein–coupled receptor
IP3	1,4,5-trisphosphate
IP3R	IP3 receptor
L	Lipid-like density
LTCC	L-type calcium channels
MD	Molecular dynamics
MetS	Metabolic syndrome
NGF	Nerve growth factor
NOX2	NADPH oxidase 2
nSOC	Neuronal SOC
PAH	Pulmonary arterial hypertension
PIP2	Phosphatidylinositol 4,5-bisphosphate
PKC	Protein kinase C
PLC	Phospholipase C
PM	Plasma membrane
PTEN	Phosphatase and Tensin Homolog Deleted from Chromosome 10
ROS	Reactive oxygen species
SOCE	Store-operated Ca^2+^ entry
TAC	Transverse aortic constriction
TG	Thapsigargin
TMD	Transmembrane domain
TRPC	Transient receptor potential canonical
VSMC	Vascular smooth muscle cell
